# Deep learning algorithm performs similarly to radiologists in the assessment of prostate volume on MRI

**DOI:** 10.1007/s00330-022-09239-8

**Published:** 2022-11-12

**Authors:** Erik Thimansson, J. Bengtsson, E. Baubeta, J. Engman, D. Flondell-Sité, A. Bjartell, S. Zackrisson

**Affiliations:** 1 Department of Translational Medicine, Diagnostic Radiology, Lund University, Carl-Bertil Laurells gata 9, SE-205 02 Malmö, Sweden; 2 Department of Radiology, Helsingborg Hospital, Helsingborg, Sweden; 3 Department of Clinical Sciences, Diagnostic Radiology, Lund University, Lund, Sweden; 4 Department of Imaging and Functional Medicine, Skåne University Hospital, Malmö, Sweden; 5 Department of Imaging and Functional Medicine, Skåne University Hospital, Lund, Sweden; 6 Department of Translational Medicine, Urological Cancers, Lund University, Malmö, Sweden; 7 Department of Urology, Skåne University Hospital, Malmö, Sweden

**Keywords:** Magnetic resonance imaging, Prostate neoplasms, Deep learning, Prostate-specific antigen

## Abstract

**Objectives:**

Prostate volume (PV) in combination with prostate specific antigen (PSA) yields PSA density which is an increasingly important biomarker. Calculating PV from MRI is a time-consuming, radiologist-dependent task. The aim of this study was to assess whether a deep learning algorithm can replace PI-RADS 2.1 based ellipsoid formula (EF) for calculating PV.

**Methods:**

Eight different measures of PV were retrospectively collected for each of 124 patients who underwent radical prostatectomy and preoperative MRI of the prostate (multicenter and multi-scanner MRI’s 1.5 and 3 T). Agreement between volumes obtained from the deep learning algorithm (PV_DL_) and ellipsoid formula by two radiologists (PV_EF1_ and PV_EF2_) was evaluated against the reference standard PV obtained by manual planimetry by an expert radiologist (PV_MPE_). A sensitivity analysis was performed using a prostatectomy specimen as the reference standard. Inter-reader agreement was evaluated between the radiologists using the ellipsoid formula and between the expert and inexperienced radiologists performing manual planimetry.

**Results:**

PV_DL_ showed better agreement and precision than PV_EF1_ and PV_EF2_ using the reference standard PV_MPE_ (mean difference [95% limits of agreement] PV_DL_: −0.33 [−10.80; 10.14], PV_EF1_: −3.83 [−19.55; 11.89], PV_EF2_: −3.05 [−18.55; 12.45]) or the PV determined based on specimen weight (PV_DL_: −4.22 [−22.52; 14.07], PV_EF1_: −7.89 [−30.50; 14.73], PV_EF2_: −6.97 [−30.13; 16.18]). Inter-reader agreement was excellent between the two experienced radiologists using the ellipsoid formula and was good between expert and inexperienced radiologists performing manual planimetry.

**Conclusion:**

Deep learning algorithm performs similarly to radiologists in the assessment of prostate volume on MRI.

**Key Points:**

*• A commercially available deep learning algorithm performs similarly to radiologists in the assessment of prostate volume on MRI.*

*• The deep-learning algorithm was previously untrained on this heterogenous multicenter day-to-day practice MRI data set.*

**Supplementary Information:**

The online version contains supplementary material available at 10.1007/s00330-022-09239-8.

## Introduction

Prostate volume (PV) is an important parameter in the workup of benign and malignant prostate diseases [1–3]. Combining the prostate specific antigen (PSA) value and PV yields the PSA density (PSAD) [4–6]. A higher PSAD, often using a threshold of 0.15 ng/ml^2^, indicates a higher risk of prostate cancer [7, 8]. PSAD is an increasingly important factor in making decisions on which patients will undergo biopsies [4]. Last years’ paradigm shift towards “MRI first” [9, 10], meaning that the patient undergoes magnetic resonance imaging (MRI) before biopsies, has made MRI a cornerstone for determining PV. PIRADS [11] recommends the ellipsoid formula method for determined PV; the radiologist measures the prostate height, depth, and width. This ellipsoid formula (EF) is considered the gold standard and has been shown to be relatively accurate [12], but it has some limitations. Use of the EF is time-consuming, reader-dependent, and prone to multiplication errors due to the prostate not being a symmetrical geometrical ellipsoid body, posing anatomical challenges delineating the apex and ventral portion [4, 13, 14]. The most accurate method for assessing PV on MRI is manual planimetry [12, 15, 16], in which the radiologist uses external software to manually outline the prostate boundaries on T2-weighted MRI in three planes. However, manual planimetry is too time-consuming to be a realistic alternative in clinical routine [17].

In the last few years, there has been growing interest in the development of artificial intelligence (AI)–based algorithms in radiology. The most commonly used AI method in imaging is deep learning convolutional neural network–based algorithms [18]. Several studies have shown good performance of AI for automated assisted PV assessment [18–20], but questions remain about external validation, generalizability, and how well the algorithm performs in a different clinical context with heterogenous data. The number of algorithms cleared by the U.S. Food and Drug Administration (FDA) continues to grow [21], though with increasing concern regarding algorithms’ true performance in the clinical setting beyond the institutions in which they were trained and validated. To subsidize to these issues, we designed this multicenter, multi-scanner study. We used a proprietary, commercially available deep learning–based system [22] not previously exposed to the data set. To make the comparison between PV methods as comprehensive as possible, we compared them to PVs from transrectal ultrasound and prostatectomy specimens [23–25].

The primary aim of this study was to assess whether a previously unexposed deep learning algorithm can replace the PI-RADS 2.1-based ellipsoid formula for calculating the PV from a heterogenous data set from prostate MRI.

The secondary aims were to evaluate the inter-reader agreement between two radiologists using PI-RADS 2.1–based ellipsoid formula and between experienced and inexperienced radiologists performing manual planimetry.

## Materials and methods

### Study design and population

**Fig. 1:**
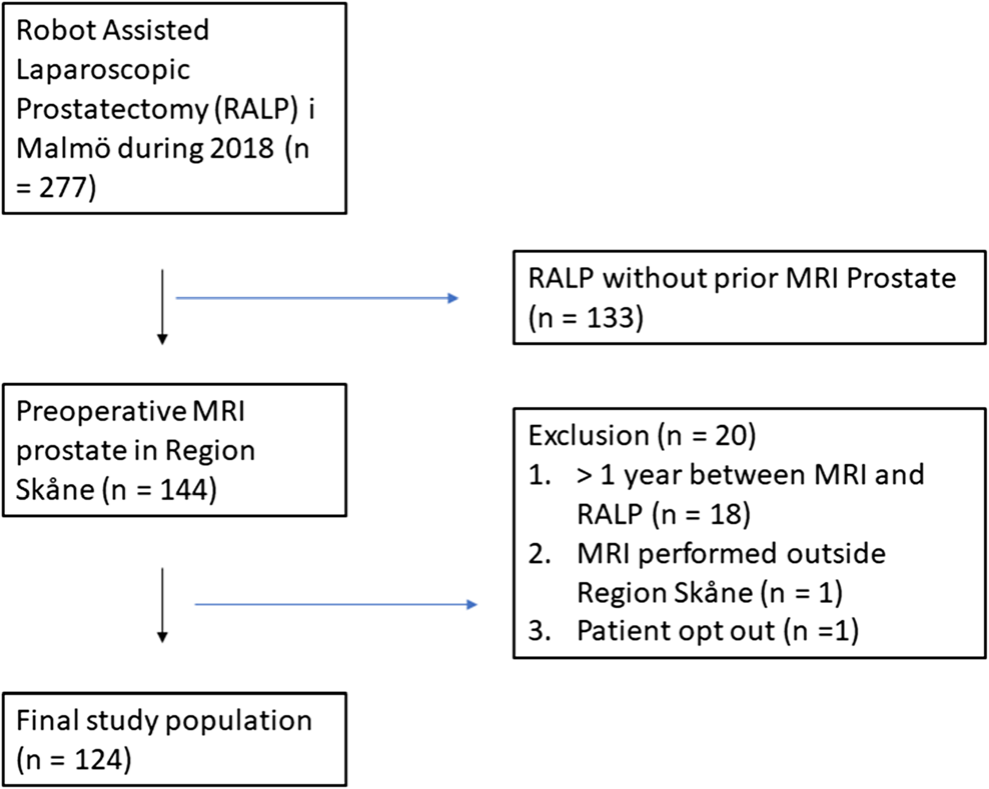
Study cohort

This retrospective multicenter study was approved by the local ethics review committee at Lund University (entry no. 2014-886) and the Swedish Ethical Review Authority (entry no. 2019-03674). All consecutive patients who underwent robot-assisted radical prostatectomy at Malmö University Hospital in 2018 were identified and assessed for eligibility. Patients were included if they had undergone MRI of the prostate less than 1 year before the surgery. Two patients were excluded due to MRI at a hospital outside our health care region and patient withdrawal, resulting in the inclusion of 124 patients in the study. The data collection algorithm is presented in Fig. 1. Eight different PVs were calculated per patient (Table 1).

**Table 1:** Descriptions and abbreviations of prostate volumes

Abbreviation	Name	Description
PV_MPE_	Prostate volume by manual planimetry, expert	Radiologist experienced in manual planimetry
PV_MPU_	Prostate volume by manual planimetry, inexperienced	Radiologist inexperienced in manual planimetry
PV_EF1_	Prostate volume by ellipsoid formula radiologist 1	Radiologist
PV_EF2_	Prostate volume by ellipsoid formula radiologist 2	Radiologist
PV_TRUS_	Prostate volume by transrectal ultrasound	Urologist
PV_DL_	Prostate volume by deep learning	Deep learning algorithm
PV_SD_	Prostate volume based on specimen dimensions	Measurements by pathology department staff
PV_SW_	Prostate volume based on specimen weight	Measurements by pathology department staff

### MRI technique

The MRI scans were performed at seven different hospitals using eight different scanners, comprising seven different scanner models from two vendors, two different field strengths (1.5 and 3T), and two different T2 transaxial (axial) slice angulations. Different imaging acquisition parameters were used at different sites according to local routines. All protocols included transversal, coronal, and sagittal T2-weighted turbo spin-echo images, which were used for ellipsoid formula calculations, and the T2 axial, which was used for manual planimetry and deep learning contouring. The parameters for the T2 axial are listed in Supplemental Table 1 in electronic supplementary material.

### Prostate volume calculations from imaging

All MRI exams were retrospectively read by three consulting radiologists (E.T., J.B., and J.E.) with at least 5 years of experience with MRI prostate. One radiologist (J.E.; highly experienced in manual planimetry from 5 years of fusion biopsy planning at a tertiary referral center) performed manual planimetry by manually tracing the prostate boundaries on the T2 image in three planes using external software (MIM Software, Inc.), blinded to all other volume calculations. This volume, PV_MPE_, was used as the reference standard, and time consumption for the whole workflow for this manual planimetry was measured on part of the exams (14 patients).

**Fig. 2:**
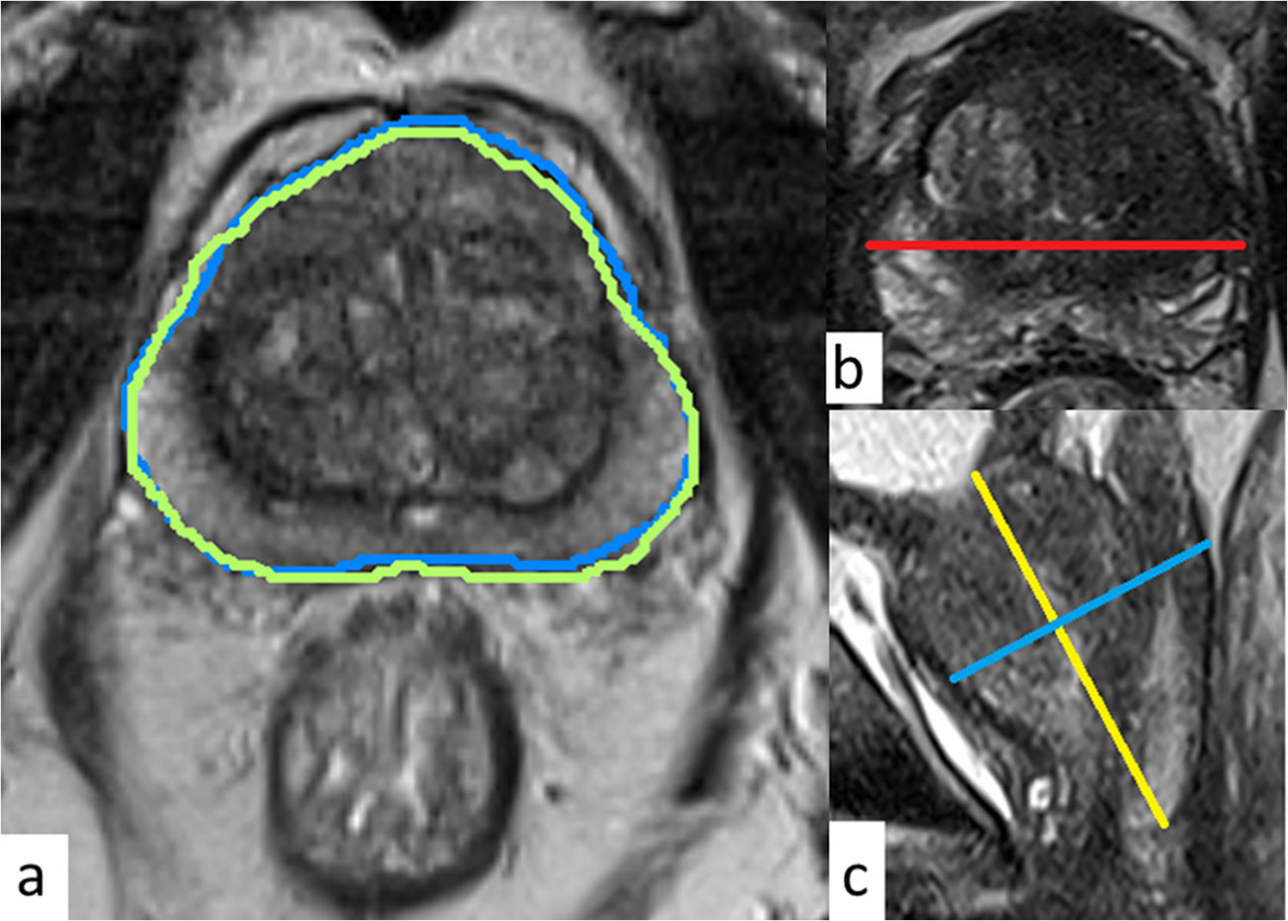
**a** One out of approximately 30 T2 transaxial images with the prostate contour outlined by planimetry (blue line, expert radiologist [PV_MPE_]; green line, deep learning algorithm [PV_DL_]). **b** T2 transaxial and **c** T2 sagittal images with measurements for the ellipsoid formula (PV_EF1_, PV_EF2_; red line, transverse diameter; yellow line, longitudinal diameter; blue line, anterio-posterior diameter)

Another radiologist (E.T.) inexperienced in manual planimetry performed manual planimetry (PV_MPU_) using the same software, but on a different server to secure blinding. Two radiologists (E.T. and J.B.) calculated the PVs using the ellipsoid formula method ([width × depth × height] × [π/ 6]) according to PIRADS [11], shown in Fig. 2b, c. This was done independently and blinded for all other PVs; these volumes are abbreviated PV_EF1_ and PV_EF2_.

PVs were also calculated from transrectal ultrasound according to the ellipsoid formula. The ultrasounds were obtained during a routine clinical visit to the urology department and collected from the patient records.

### Deep learning algorithm

The algorithm used is a proprietary commercially available product (AI-Rad Companion Prostate MR VA20A_HF02, Siemens Healthcare AG), a machine learning deep learning algorithm that uses a convolutional neural network deep image to image (DI2IN) network. The algorithm was not previously exposed to the images in the current study cohort and whole gland segmentation was performed on non-annotated T2 axial images as described by Yang [26]. The contours and volume calculations (PV_DL_) were exported back to and saved in the Picture Archive and Communications System (Sectra IDS7). The results were presented as burnt-in contours and PV as text. Contours outlined by deep learning algorithm and expert radiologist are shown in Fig. 2a.

### Prostatectomy specimens

Prostatectomy specimens were processed and prepared according to international standard pathological procedures [27], embedding all material, including seminal vesicles and extraprostatic tissue. Specimen dimensions in three planes and weight were obtained from pathology reports. Specimen volume was calculated using the ellipsoid formula (PV_SD_) [12]. Specimen weight and the prostate density coefficient (1.05 g /mL) [24, 25] were also used to calculate PV (PV_SW_).

### Statistical analysis

Descriptive statistics were used to describe the study cohort as medians and ranges. A box plot was used to present the distribution of volumes according to the different methods.

Agreement between volume measurement methods was evaluated using Bland Altman plots. First, we compared deep learning and ellipsoid formula-determined volumes (i.e., PV_DL_ vs. PV_EF1_ and PV_EF2_) in relation to the manual planimetry standard (i.e., PV_MPE_). Second, we performed a sensitivity analysis comparing the deep learning and ellipsoid formula-determined volumes in relation to the volumes determined based on specimen weight (i.e., PV_SW_).

Using Bland Altman plots, inter-reader agreement was analyzed between an experienced and inexperienced radiologist performing manual planimetry (i.e., PV_MPE_ vs. PV_MPU_) and between two radiologists using the ellipsoid formula for volume assessment (i.e., PV_EF1_ vs. PV_EF2_). Inter-reader agreement was also measured for the latter using the intraclass correlation (ICC). The formula for random effects, absolute agreement, and single rater measurements was used. The paired *t*-test was used to compare the mean differences between the experienced and inexperienced radiologist performing manual planimetry and paired *t*-test with Bonferroni correction was used to compare volume methods. All statistical analyses were performed in R version 4.0.2 [28].

## Results

The cohort consisted of 124 patients with a median age of 66 years (range 45–76 years) and median preoperative PSA of 6.90 μg/L (min 0.88; max 39).

**Fig. 3:**
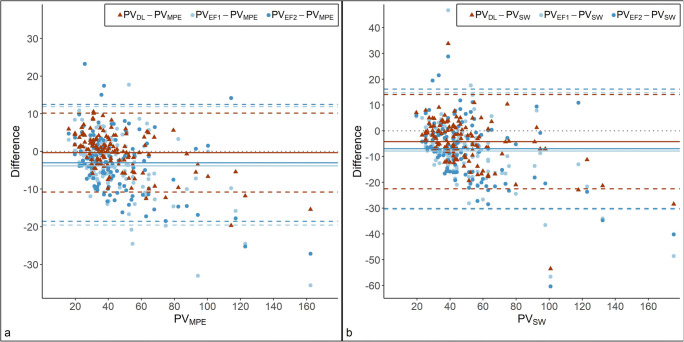
Bland Altman plot comparing two methods of measuring prostate volume: automated deep learning (PV_DL_) and manual ellipsoid formula performed by two radiologists (PV_EF1_ and PV_EF2_). **a** Prostate volumes from manual planimetry by an experienced radiologist (PV_MPE_) were used as a reference standard. **b** Prostate volumes from prostatectomy specimen weight (PV_SW_) were used as a reference standard. The solid lines represent the mean difference between methods and the dashed lines limits of agreement, calculated as the mean difference ± 1.96SD. The dotted line represents no difference between methods

As shown in Fig. 3a, the observed mean difference between PV_DL_ and PV_MPE_ was lower than the observed mean difference between PV_EF_ and PV_MPE_ (mean difference [95% limits of agreement] PV_DL_: −0.33 mL [−10.80; 10.14 mL], PV_EF1_: −3.83 mL [−19.55; 11.89 mL], PV_EF2_: −3.05 mL [−18.55; 12.45 mL]). The limits of agreement were slightly narrower for PV_DL_ than PV_EF_, indicating better precision.

A sensitivity analysis using PV_SW_ as the reference standard is presented as a Bland Altman plot in Fig. 3b. The mean difference (bias) was lower for PV_DL_ than PV_EF1_ or PV_EF2_ and the corresponding 95% limits of agreement (mean + 1.96 SD and mean −1.96 SD) narrower for PV_DL_ than PV_EF1_ or PV_EF2_ using PV_MPE_ as the reference standard (mean difference [95% limits of agreement] PV_DL_: −4.22 mL [−22.52; 14.07 mL], PV_EF1_: −7.89 mL [−30.50; 14.73 mL], PV_EF2_: −6.97 mL [−30.13; 16.18 mL]). In both Bland Altman plots (Fig. 3a, b), there was a tendency of deep learning and the ellipsoid formula to underestimate volumes of enlarges prostates compared to both manual planimetry and specimen weight, but deep learning seemed to underestimate the large volumes to a lesser extent than the ellipsoid formula.

**Fig. 4:**
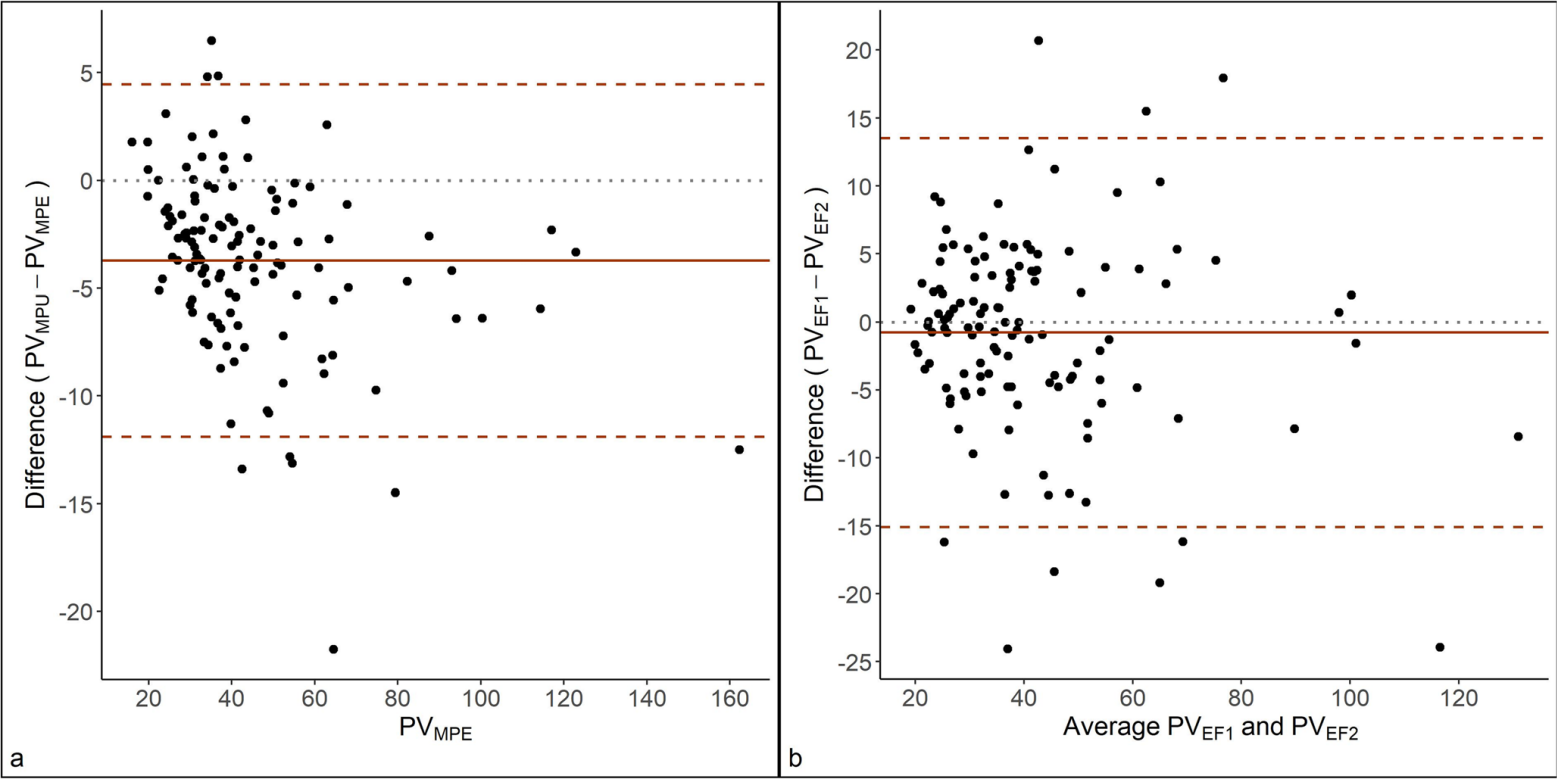
**a** Bland Altman plot comparing manual planimetry by experienced (PV_MPE_) and inexperienced radiologists (PV_MPU_) for measuring prostate volume. **b** Bland Altman plot comparing prostate volumes measured by the ellipsoid formula by two radiologists. The solid lines represent the mean difference between methods and the dashed lines limits of agreement, calculated as the mean difference ± 1.96SD. The dotted line represents no difference between methods

The inter-reader agreement between the two radiologists performing manual planimetry is shown in Fig. 4a, indicating that the inexperienced reader systematically calculated lower volumes than the experienced reader, but with better precision than between the two radiologists using the ellipsoid formula (mean difference [95% limits of agreement] PV_MPE_ vs. PV_MPU_: −3.73 mL [−11.90; 4.45 mL], *p*<0.001, paired *t*-test; 95% confidence interval [CI] −4.47; −2.99). The inter-reader agreement between PV_EF1_ and PV_EF2_ is shown in Fig. 4b. The mean difference (95% limits of agreement) between reader 1 and reader 2 was −0.78 mL (−15.08; 13.51 mL). The ICC (95% CI) was 0.93 (0.96; 0.953).

**Fig. 5:**
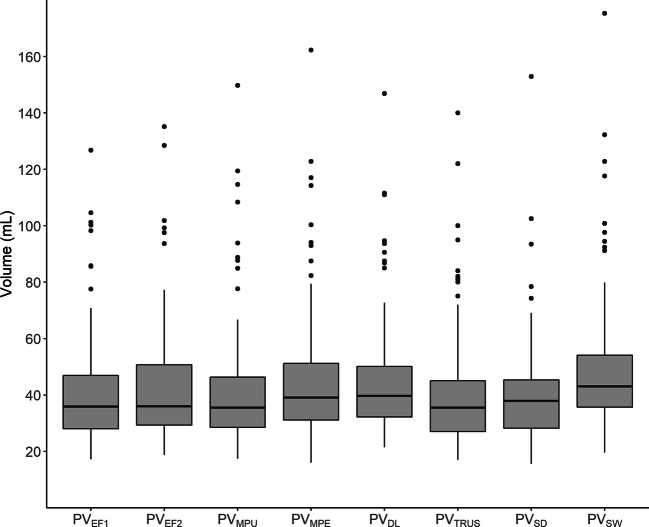
Distribution of volumes according to eight different methods for measuring prostate volume. PV_EF1_ = prostate volume by ellipsoid formula radiologist 1, PV_EF2_ = prostate volume by ellipsoid formula radiologist 2, PV_MPU_ = prostate volume by manual planimetry by inexperienced radiologist, PV_MPE_ = prostate volume by manual planimetry by experienced radiologist, PV_DL_= prostate volume by deep learning, PV_TRUS_= prostate volume by transrectal ultrasound, PV_SD_ = prostate volume based on specimen dimensions, PV_SW_ = prostate volume based on specimen weight. The center line denotes the median value, the box contains the 25th to 75th percentiles of dataset. The whiskers mark the 5th and 95th percentiles, and values beyond these upper and lower bounds are considered outliers, marked with dots

Table 2 and the box and whisper plot in Fig. 5 show the mean, median, minimum, and maximum values for all compared volumes. Supplemental Table 2 in electronic supplementary material shows which of the combinations of the compared volumes statistically significant differences were found. Timed observations for planimetry by an experienced reader were recorded in 14/124 patients, and mean time consumption per case was 8 min 4 s.

**Table 2:** Eight prostate volume estimates

Measurement	Overall (*N* = 124)
PV_EF1_	
Mean (SD)	41.2 (19.4)
Median [min, max]	35.8 [17.2, 127]
PV_EF2_	
Mean (SD)	42.0 (20.5)
Median [min, max]	36.0 [18.7, 135]
PV_MPU_	
Mean (SD)	41.3 (21.4)
Median [min, max]	35.5 [17.4, 150]
PV_MPE_	
Mean (SD)	45.0 (22.5)
Median [min, max]	39.1 [16.0, 162]
PV_DL_	
Mean (SD)	44.7 (19.9)
Median [min, max]	39.7 [21.4, 147]
PV_TRUS_	
Mean (SD)	40.2 (20.1)
Median [min, max]	35.5 [17.0, 140]
Missing	2 (1.6%)
PV_SD_	
Mean (SD)	39.6 (17.8)
Median [min, max]	37.9 [15.6, 153]
PV_SW_	
Mean (SD)	48.9 (23.1)
Median [min, max]	43.1 [19.5, 175]
Missing	1 (0.8%)

## Discussion

This study shows that the PVs obtained from MRI using a commercially available deep learning algorithm have better agreement and precision with two reference standard volumes than the currently recommended gold standard method performed by a radiologist. There was good inter-reader agreement between two experienced radiologists using the ellipsoid formula. Furthermore, this study indicates a small but significant mean difference (low bias) with good precision when evaluating inter-reader agreement between experienced and inexperienced radiologists performing manual planimetry.

In 2013, Turkbey et al [29] showed highly accurate PV estimates by a non-commercially available deep learning algorithm compared to specimen volumes in 99 patients using correlation and Dice similarity coefficients [30] (Pearson coefficient 0.88–0.91, DICE 0.89). They reported no difference compared to manual planimetry. This result is similar in the present study, as the bias between the deep learning algorithm and manual planimetry was close to zero (−0.33 mL). In 2018, Bezinque et al [12] showed in 99 patients that the most reliable method for PV measurement was manual planimetry by an inexperienced reader (ICC 0.91), followed by ellipsoid formula on MRI (ICC 0.9), compared to manual planimetry by an experienced reader. The authors concluded that automated segmentations (ICC 0.38) made by commercially available software must be individually assessed for accuracy, contradictory to the results of the present study. The amount of training data available for algorithms is rapidly increasing, which may explain the difference in performance in the current study and older studies. In 2020, Lee et al [19] evaluated a non-commercially available deep learning algorithm on a 330 MRI (260 training and 70 test case sets) using manual planimetry as the reference standard. The authors concluded that the algorithm provided reliable PV estimates (ICC 0.90) compared to those obtained with the ellipsoid formula (ICC 0.92). The mean error between algorithm and manual planimetry was 2.5 mL (0.33 mL in our study) and 3.3 mL between ellipsoid formula and manual planimetry (3.05–3.83 mL in our study). In 2021, Salvaggio et al [31] used manual planimetry as the reference standard when they evaluated two deep learning algorithms for prostate segmentation in a cohort of 103 patients. The authors concluded that the presence of median lobe enlargement may lead to overestimation by the ellipsoid formula, recommending a segmentation method. In 2021, Cuocolo et al [32] compared different deep learning algorithms in 204 patients (99 training sets and 105 test sets) using manual planimetry as the reference standard. The efficient neural network (ENet) showed the best performance with the lowest relative volume difference compared to reference standard.

In our study, the mean difference compared to specimen weight volumes (using the specimen gravity formula) was less than zero for both the deep learning algorithm and the two ellipsoid formula measurements (−4.22 mL, −7.89 mL, and −6.97 mL, respectively), indicating a systematic underestimation in line with Bezinque et al [12]. On the other hand, Paterson et al [13] showed that the ellipsoid formula overestimated by a mean 1.4 mL and Lee et al [19] that it overestimated by 2.4%. The discrepant results may be related to a different proportion of cases with median lobe hypertrophy or measurement difficulties and inconsistencies when dealing with extraprostatic tissue.

As described by Salvaggio [31], the correlation between PVs obtained from MRI or radical prostatectomy specimens is dependent on the PV itself, a tendency that we also saw in our material, with overestimation of small prostate gland volumes and underestimation of large prostate gland volumes.

Our study showed good inter-reader agreement between two radiologists estimating PVs with the ellipsoid formula according to PIRADS v 2.1 (mean difference −0.78) and ICCs that were in line with two previous studies [17, 33].

Compared to the present study, Bezinque [12] reported somewhat better agreement between experienced and inexperienced radiologists, with a mean difference of −1.00 mL and ICC of 0.91. Differences in study cohorts and design make comparisons between the studies difficult. A small amount of bias can be acceptable as long as the precision is good (as shown by narrow limits of agreement), which seems to be the case with our results.

In this study, we evaluated the agreement between deep learning and ellipsoid formula-determined volumes against two different reference standards. The first evaluation against manual planimetry as the reference standard which was also performed by Cuocolo, Lee, and Bezinque [12, 19, 32]. To avoid the results relying mainly on the similar methodology between deep learning and manual planimetry (i.e., whole gland segmentation by outer contours), we performed a sensitivity analysis by changing the reference standard to the PV based on specimen weight, as used by Turkbey, Mazaheri, and Bulman [17, 23, 29]. The results show that, for both reference standards, the deep learning PVs had lower bias and narrower limits of agreement, meaning better precision than the ellipsoid formula volumes.

To compare the agreement between methods for PV measurement, we based our statistical analysis and visualizations on Bland Altman plots. Several previous studies comparing methods for measuring PV [12, 17, 29] have used different statistical methods based on correlation, and several studies have used DICE similarity coefficients [19, 32]. Correlation coefficients tell us about the linear relationship but not about the agreement, which is indicated by the limits of agreement. In our opinion, the DICE coefficients do not add value to the comparison of methods as evaluated in this study, but they play a key role when studying the quality of outer contour delineation for MRI/ultrasound fusion biopsy and brachytherapy planning. This is the topic of a planned future study by our research group.

Our study has several strengths. The deep learning algorithm was previously unexposed to the data and, to the best of our knowledge, the test set was larger than in previous studies [12, 17, 19, 29, 32]. To reflect a true clinical context, we used a heterogenous MRI data set with a multicenter, multi-scanner setup. We used a proprietary commercially available deep learning algorithm, whereas Lee [19] evaluated a non-commercially available 3D deep learning convolutional neural network. Cuocolo [32] compared three different deep learning networks (ENet, ERFNet, and U-net), concluding that deep learning networks can accurately segment the prostate and Enet performed best. In this study, all MRI exams were resampled to isotropic voxel size and to identical matrix resolution to facilitate the deep learning segmentation and, in our study, no resampling was necessary despite variations in MRI protocols.

We investigated the possibility to perform a validation of the deep learning algorithm on publicly available datasets. The available public datasets with manual planimetry as reference standard [34, 35] had been included in early training of the algorithm, why a performance test could not be performed as data sets for training, validation, and testing must always be unique and separated. It is reasonable to believe this applies also for other commercially available deep learning algorithms, which emphasizes the importance of study designs like the current one, using heterogeneous clinically relevant datasets when testing the robustness of AI models.

This study has some limitations. Firstly, only one algorithm was tested. In addition, although the MRI data set is heterogeneous, one vendor is dominant, which is also the same company behind the evaluated algorithm. Furthermore, the cohort was only prostatectomy patients, which does not reflect the clinical setting, where a larger variation of both cancer and non-cancer patients is scanned. The evaluated algorithm does not offer sub-segmentation of transition and peripheral zone separately. Sub-segmentation would enable more elaborated density calculation, like prostate volume index and transition zone PSAD [36, 37]. Those measurements can add prognostic value for use in a clinical setting with mixed patients (with cancer, no cancer and inflammation). However, in this current study, with cancer cases only, they play a minor role. We plan on future studies dealing with these limitations by evaluating several algorithms with different scanner vendors and a more heterogeneous patient group. A future study should ideally be designed as a non-inferiority study with power calculation for adequate cohort size. Only one experienced radiologist performed manual planimetry. However, it is known there is an interreader variability in manual planimetry [38]. We tried to take this into consideration by letting a second radiologist (less experienced) also perform manual planimetry and via performing the sensitivity analysis with specimen volumes.

### Clinical implications

The number of U.S. FDA-approved commercial AI/deep learning algorithms is rapidly increasing [21], but there is a concern regarding the challenges accompanying the application of those algorithms in the clinical routine. There is a need for structured monitoring and follow-up when we start using those algorithms in day-to-day practice.

To the best of our knowledge, no earlier studies have evaluated a proprietary commercially available deep learning algorithm on such a heterogeneous MRI data set as in this study. The current study setup with a multicenter, multi-scanner, multi-parameter protocol resembles the true clinical situation in a diversified national or international setting.

## Conclusion

A deep learning algorithm is at least as good as the PI-RADS 2.1–based ellipsoid formula for assessing PV when compared to in vivo and ex vivo reference standards. This is a promising step towards algorithms helping reallocate radiologist resources towards more complex work tasks than manually measuring PVs.

## Supplementary Information

ESM 1 (DOCX 41 kb)

ESM 2 (XLSX 13 kb)

## Abbreviations

AI: Artificial intelligence
ICC: Intraclass correlation
MRI: Magnetic resonance imaging
PIRADS: Prostate Imaging Reporting and Data System
PSA: Prostate-specific antigen
PSAD: Prostate-specific antigen density
PV: Prostate volume
PVDL: Prostate volume obtained from deep learning algorithm
PVEF: Prostate volume obtained from radiologist using ellipsoid formula
PVMPE: Prostate volume obtained from manual planimetry by expert radiologist
PVMPU: Prostate volume obtained from manual planimetry by unexperienced radiologist
PVSD: Prostate volume obtained from specimen dimensions
PVSW: Prostate volume obtained from specimen weight
PVTRUS: Prostate volume obtained from transrectal ultrasound
EF: PI-RADS 2.1 based ellipsoid formula

## Declarations

### Guarantor

The scientific guarantor of this publication is Professor Sophia Zackrisson.

### Statistics and biometry

Andrea Dahl Sturedahl kindly provided statistical advice for this manuscript.

No complex statistical methods were necessary for this paper.

### Methodology

• retrospective

• diagnostic or prognostic study

• multicenter study
