# Publisher Correction: Deep learning algorithm performs similarly to radiologists in the assessment of prostate volume on MRI

**DOI:** 10.1007/s00330-022-09308-y

**Published:** 2022-12-06

**Authors:** Erik Thimansson, J. Bengtsson, E. Baubeta, J. Engman, D. Flondell-Sité, A. Bjartell, S. Zackrisson

**Affiliations:** 1 Department of Translational Medicine, Diagnostic Radiology, Lund University, Carl-Bertil Laurells gata 9, SE-205 02 Malmö, Sweden; 2 Department of Radiology, Helsingborg Hospital, Helsingborg, Sweden; 3 Department of Clinical Sciences, Diagnostic Radiology, Lund University, Lund, Sweden; 4 Department of Imaging and Functional Medicine, Skåne University Hospital, Malmö, Sweden; 5 Department of Imaging and Functional Medicine, Skåne University Hospital, Lund, Sweden; 6 Department of Translational Medicine, Urological Cancers, Lund University, Malmö, Sweden; 7 Department of Urology, Skåne University Hospital, Malmö, Sweden

**Publisher Correction: European Radiology**

10.1007/s00330-022-09239-8

The original version of this article, published on 12 November 2022, unfortunately contained a mistake. The author’s name Erik Thimansson was incorrectly given as ‘Erick Thimansson’. The original article has been corrected.

