# Supplementary material for: Deep learning algorithm performs similarly to radiologists in the assessment of prostate volume on MRI
Source: Eur Radiol. 2022 Nov 12;33(4):2519–28. doi: 10.1007/s00330-022-09239-8 (PMC10017633; doi:10.1007/s00330-022-09239-8)
Supplement: Supplementary file 1 — (DOCX 41 kb) [file 330_2022_9239_MOESM1_ESM.docx]

| **Method 1** | **Method 2** | **Mean difference** | **Bonferroni corrected p-values** |
| --- | --- | --- | --- |
| EF1 | EF2 | -0.785 | 1.00 |
| EF1 | MPU | -0.108 | 1.00 |
| EF1 | MPE | -3.83 | <0.0001 |
| EF1 | DL | -3.51 | <0.0001 |
| EF1 | TRUS | 0.999 | 1.00 |
| EF1 | SD | 1.63 | 1.00 |
| EF1 | SW | -7.89 | <0.0001 |
| EF2 | MPU | 0.68 | 1.00 |
| EF2 | MPE | -3.05 | 0.0010 |
| EF2 | DL | -2.72 | 0.004 |
| EF2 | TRUS | 1.77 | 1.00 |
| EF2 | SD | 2.41 | 0. 969 |
| EF2 | SW | -6.97 | <0.0001 |
| MPU | MPE | -3.73 | <0.0001 |
| MPU | AI | -3.40 | <0.0001 |
| MPU | TRUS | 1.13 | 1.00 |
| MPU | SD | 1.73 | 1.00 |
| MPU | SW | -7.56 | <0.0001 |
| MPE | DL | 0.33 | 1.00 |
| MPE | TRUS | 4.89 | <0.0001 |
| MPE | SD | 5.46 | <0.0001 |
| MPE | SW | -3.98 | 0.0001 |
| DL | TRUS | 4.54 | <0.0001 |
| DL | SD | 5.13 | <0.0001 |
| DL | SW | -4.22 | <0.0001 |
| TRUS | SD | 0.633 | 1.00 |
| TRUS | SW | -9.00 | <0.0001 |
| SD | SW | -9.37 | <0.0001 |

Supplemental table 2: Paired t-test with Bonferroni correction for multiple comparisons (28 comparisons). p<0.05 in rows filled with blue.
